# Assessing the Physiological Relevance of Cough Simulators for Respiratory Droplet Dispersion

**DOI:** 10.3390/jcm9093002

**Published:** 2020-09-17

**Authors:** Shiv H. Patel, Wonjun Yim, Anupam K. Garg, Sahil H. Shah, Jesse V. Jokerst, Daniel L. Chao

**Affiliations:** 1 Simulation Training Center, University of California, San Diego, La Jolla, CA 92093, USA; shp093@ucsd.edu; 2 Division of Biological Sciences, University of California, San Diego, 9500 Gilman Drive, La Jolla, CA 92093, USA; 3 Department of Material Science and Engineering, University of California, San Diego, La Jolla, CA 92093, USA; woyim@eng.ucsd.edu (W.Y.); jjokerst@eng.ucsd.edu (J.V.J.); 4 School of Medicine, University of California, San Diego, La Jolla, CA 92093, USA; akgarg@health.ucsd.edu (A.K.G.); sas032@health.ucsd.edu (S.H.S.); 5 Department of Radiology, University of California, San Diego, 9500 Gilman Drive, La Jolla, CA 92093, USA; 6 Department of Nanoengineering, University of California, San Diego, La Jolla, CA 92093, USA; 7 Viterbi Family Department of Ophthalmology, Shiley Eye Institute, University of California, San Diego, La Jolla, CA 92093, USA

**Keywords:** simulation, COVID-19, viral surrogates, infectious droplets, respiratory droplets, dispersion modeling, cough, breath, GloGerm, atomizer

## Abstract

Various breathing and cough simulators have been used to model respiratory droplet dispersion and viral droplets, in particular for SARS-CoV-2 modeling. However, limited data are available comparing these cough simulations to physiological breathing and coughing. In this study, three different cough simulators (Teleflex Mucosal Atomization Device Nasal (MAD Nasal), a spray gun, and GloGerm^TM^ MIST) that have been used in the literature were studied to assess their physiologic relevance. Droplet size, velocity, dispersion, and force generated by the simulators were measured. Droplet size was measured with scanning electron microscopy (SEM). Slow-motion videography was used to 3D reconstruct and measure the velocity of each simulated cough. A force-sensitive resistor was used to measure the force of each simulated cough. The average size of droplets from each cough simulator was 176 to 220 µm. MAD Nasal, the spray gun, and GloGerm^TM^ MIST traveled 0.38 m, 0.89 m, and 1.62 m respectively. The average velocities for the MAD Nasal, spray gun, and GloGerm^TM^ MIST were 1.57 m/s, 2.60 m/s, and 9.27 m/s respectively, and all yielded a force of <0.5 Newtons. GloGerm^TM^ MIST and the spray gun most closely resemble physiological coughs and breathing respectively. In conclusion, none of the simulators tested accurately modeled all physiologic characteristics (droplet size, 3-D dispersion velocity, and force) of a cough, while there were various strengths and weaknesses of each method. One should take this into account when performing simulations with these devices.

## 1. Introduction

**Figure 1:**
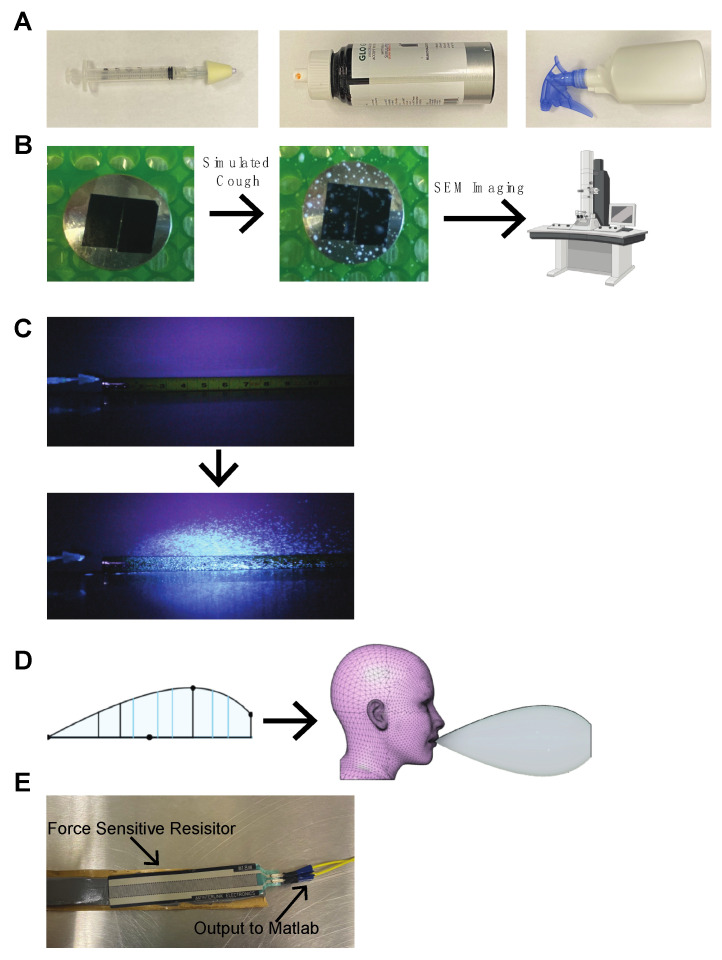
Overview of Experimental Strategy. (**A**) Images of the three cough simulators: MAD Nasal, GloGerm^TM^ MIST, and the spray gun (left to right). (**B**) A conductive mounting tab with 8 mm carbon tape is shown before and after collecting droplets. (**C**) Still frames from the video used to quantify simulated cough velocity for MAD Nasal before and after a simulated cough. (**D**) The 2D reconstruction for a MAD Nasal simulated cough and a profile of the resulting 3D reconstruction on Fusion 360. (**E**) A photograph of the force-sensing resistor used for the force measurement experiment.

The COVID-19 global pandemic, caused by the spread of SARS-CoV-2, has exposed fundamental gaps in our knowledge of viral spread. It is reported that two of the primary modes of spread are via respiratory droplets of >5–10 µm and aerosolized matter of <5 µm, however, there are many numerical studies emphasizing transmission via respiratory droplets to develop and inform best practices for personal protective equipment [1,2,3,4,5,6]. Therefore, here we focus on respiratory droplet spread rather than aerosol matter. We do note, however, that aerosol spread is an important aspect of transmission, and can spread over large areas over time as displayed by larger scale studies that analyze atmospheric aerosol behavior [7,8]. In order to develop and inform best practices for personal protective equipment, various simulations of respiratory and aerosolized droplet dispersion have been conducted. However, it is unclear which of these methods of simulation, if any, approximate physiologic breathing or coughing [9,10,11,12,13,14,15,16,17,18,19,20,21]. This is an important issue as data from these experiments are often used to inform guidelines for personal protective equipment (PPE) in the COVID-19 era. In order to validate cough and breathing simulators intended to measure respiratory droplet spread, we evaluated several easily accessible and low-cost cough simulators (Teleflex Mucosal Atomization Device Nasal (MAD Nasal), spray gun, and GloGerm^TM^ MIST pressurized canister) that have been used in the literature that utilize UV-visible surrogates for their physiologic relevance by assessing respiratory droplet size, velocity, spread, and force. MAD Nasal is a commonly used atomization device that has a 5 mL syringe attached to a cone consisting of a filter that turns the liquid into a mist as it flows through. The spray gun is a conventional spray bottle, such as those that hold glass cleaners, with a trigger to spray a mist of liquid. GloGerm^TM^ MIST is a pressurized aerosol canister that contains a solution of UV visible GloGerm^TM^ that turns into a mist upon exiting the nozzle (Figure 1A).

Several studies have used complex setups that require the use of cell culture media, a micropump nebulizer, or optical particle counter [10,11]. However, given their complexity, low-cost setups with a UV-visible surrogate have been widely utilized [9,12,13,14,15,16,17,18,19,20,21]. One procedure that was investigated was direct laryngoscopy intubation with and without an “intubation box” for protection against viral droplets and aerosol [12]. The simulation was done using an explosive balloon filled with fluorescent dye. However, the authors noted that there was no analysis done behind the physiological accuracy of their simulation. Numerous studies have utilized the Teleflex MAD Nasal atomizer (referred to as MAD Nasal hereafter), which is commonly used clinically to deliver drugs trans-nasal in an atomized form, to create viral droplets [13]. For example, in a recent study, MAD Nasal and GloGerm^TM^ were used to produce simulated coughs onto emergency department personnel dressed in various forms of PPE [14]. Another study that evaluated the use of a modified mask during endonasal procedures used a variant of the MAD Nasal and fluorescein to evaluate the protective power of their technology [15]. However, neither of these studies evaluated the 3D spread of the droplets from their cough simulator, nor did they evaluate the velocity of each cough or breath produced in their experiments. Another cough simulator that has been used is a pump-based spray gun attached to a conventional spray bottle to track viral droplet spread and deposition on a protective slit lamp shield during an ophthalmic clinical examination [9].

Similar techniques have also been used before the COVID-19 pandemic in order to track the spread of infectious diseases, and thus have importance and relevance beyond the scope of COVID-19. An example includes the use of GloGerm^TM^ MIST in an experiment to track the spread of droplets during donning and doffing of PPE in order to make an informed protocol to safely don and doff PPE without spreading or contracting pathogens [16]. Within the numerous studies that have used some mechanism to simulate breathing or coughing in order to track droplet spread, many are expensive, complex, and require technology not easily available to all clinicians.

Given the complexity and wide range of fidelity in cough simulators, here we investigated the physiological relevance of three widely used cough simulators. These simulators are accessible for wide use and allow analysis of droplet spread by humans after contacting a surface with viral droplets from a simulated cough. In particular, we analyze the surrogate respiratory droplet sizes, simulated cough velocity, 3D spread of each simulated cough, and the force produced by each simulated cough, and compare them to physiological conditions to define which simulators are appropriate for clinical simulation.

## 2. Experimental Section

### 2.1. Evaluation of Droplet Sizes

A solution of GloGerm^TM^ (Marlatek Inc., Brockville, ON, Canada) was used as the surrogate respiratory droplet solution for each of the simulators analyzed. In addition to the MAD Nasal (Teleflex, Wayne, PA, USA), spray gun (Whitmore, San Francisco, CA, USA) and GloGerm^TM^ MIST canister, a Phillips nebulizer was also used to evaluate droplet sizes. Simulated cough samples from each cough simulator were captured on strips of 8 mm carbon tape (TedPella, Redding, CA, USA) squares that were fixed onto conductive mounting tabs (Figure 1B). Droplet samples from each simulator were viewed under an FEI Apreo scanning electron microscope (SEM; Fisher Scientific, Waltham, MA, USA) at 70–80× magnification. SEM was then used to calibrate the size and examine the morphology of droplets at an acceleration voltage of 1kV and a current of 0.1 nA. The caliper function on the SEM interface was used to measure the diameter of each droplet seen in the 70–80× magnification frame and subsequently calculate the average droplet size for each cough simulator based on the number of droplets visible under the SEM. By reporting the droplet size ranges for each cough simulator, we aim to be consistent with how droplet size distribution analysis is reported in the literature [22,23]. The Phillips nebulizer was not used for any further tests since the droplets were not easily visible. The droplet size measurements were done independent of the velocity and force measurements.

### 2.2. Velocity Measurements

For each cough simulator, a simulated cough was delivered in front of a measuring tape under UV light. All videos were taken using a tripod-mounted Nikon D5200 digital camera. The blacklight was placed in a stationary position and video was recorded at 120 fps at 1080 p resolution. All post-capture editing was done on Adobe Premiere Pro 2020 and calibration changes of brightness and contrast were identical in all trials. Using Adobe Premiere Pro 2020, the velocity of each simulated cough was determined by following several different particles from all the replicate coughs over time and creating an average velocity for each cough simulator. The contrast and brightness were left identical in all trials. Two still frames were exported for representative images in Figure 1C. The velocity measurements were done independent of the droplet size and force measurements.

### 2.3. 3D Reconstruction of Simulated Coughs

Using the videos and photos taken during the velocity measurement experiment, each simulated cough was reconstructed in 3D by inferring data from 2D measurements. The farthest distance traveled by each simulated cough was recorded, and by adding scaled lines to stills from the video we captured, a spline was created on Fusion 360 (Autodesk, San Rafael, CA, USA). The spline was created by drawing lines orthogonal to the flow of the cough and then connecting the lines with a spline (Figure 1D). The spline was then rotated in order to create a 3D object. Using the rotation, scaled lines, and the distance traveled by the simulated cough, multiple 2D data points were used to recreate a 3D cloud or envelope. The same process was repeated for each cough simulator. Each 3D cough reconstruction was stopped at the farthest distance from the cough simulator where any visible particle was found. Using the 3D reconstruction, droplet spread and the maximum detectable 2D projected area were derived on Fusion 360. Since each simulated cough was triggered manually and there is hand motion generated between each cough for all the simulations, the 3D reconstructions were adjusted based on three trials of five spray repetitions to account for the hand motion’s influence on the simulated coughs’ 3D spread.

### 2.4. Force Measurements

A force-sensing resistor (Interlink Electronics, Camarillo, CA, USA) was connected to a myDAQ system (Texas Instruments, Dallas, TX, USA) that relayed information onto a computer where a time-dependent voltage graph was generated on Matlab (Figure 1E). When a force was added to the force-sensing resistor, the voltage within the circuit dropped and the computer recorded the voltage change on Matlab. The voltage was calibrated to force in Newtons with the addition of 100 g weights to the force-sensing resistor up to 500 g. We delivered five replicates of simulated coughs from each simulator on the force-sensing resistor along with five replicates of human breaths and five replicates of human coughs from a single individual. These activities were done in the first 25 s of the experiment and at 30 s, a 1 Newton standard weight was added to the force-sensing resistor to visualize a contrast in voltage change over time. Since there was human coughing and breathing involved, these tests were performed in isolation and all equipment and surroundings were sanitized after the experiment. The force measurements were done independent of the velocity and droplet size measurements.

## 3. Results

### 3.1. Evaluation of Droplet Sizes and Velocity 

**Figure 2:**
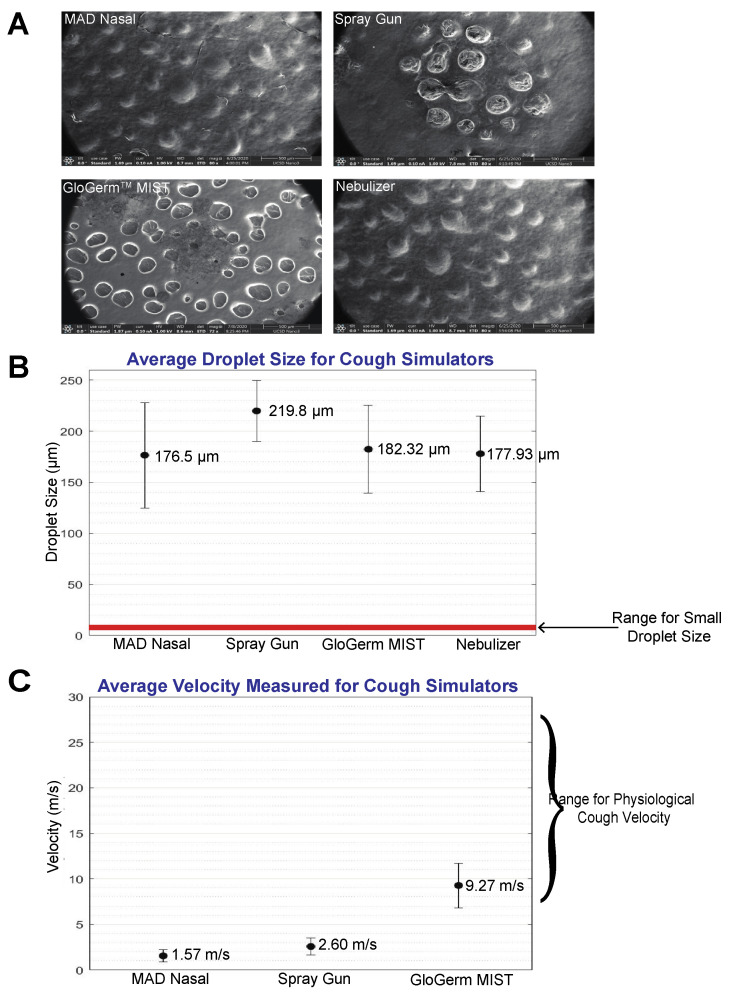
Droplet size and velocity measurements. (**A**) SEM images of droplets produced from simulated coughs produced by MAD Nasal, a spray gun, GloGerm^TM^ MIST, and a nebulizer. (**B**) A categorical plot showing the droplet sizes (µm) produced by each cough simulator. The thickness of the red line indicates the range for small respiratory droplets (5–10 µm), and error bars indicate one standard deviation. (**C**) Categorical plot showing the average measured velocity (m/s) of each cough simulator. Error bars indicate one standard deviation and brackets on the side indicate physiological coughing and breathing velocity ranges.

Our droplet size analysis revealed that the MAD Nasal, spray gun, GloGerm^TM^ MIST, and Phillips nebulizer produce droplets with 176.50 ± 51.58 µm, 219.80 ± 29.87 µm, 183.32 ± 43.01 µm, and 177.93 ± 36.88 µm (mean ± standard deviation) respectively (Figure 2A,B). Respectively, the range for the droplet sizes are 107.9 µm to 297.0 µm, 168.6 µm to 256.5 µm, 97.03 µm to 246.3 µm, and 121.4 µm to 246.2 µm. None of these droplets are within the 5–10 µm small respiratory droplet range listed in the literature, however, they are >60 µm, which is a reported threshold for large respiratory droplets in the literature. [24]. Based on prior studies, it is reported that there are two main distributions of droplets that are relevant, 1–10 µm and 100–1000 µm, and human respiratory droplets can be found to be between 1 to 2000 µm [5,25]. The mean and range for the droplets measured are distributed within the larger distribution of 100–1000 µm. The velocities of the simulated coughs from the MAD Nasal, spray gun, and GloGerm^TM^ MIST were 1.57 ± 0.66 m/s, 2.60 ± 0.93 m/s, and 9.27 ± 2.44 m/s, respectively (Figure 2C). In comparison to physiological breathing velocity, MAD Nasal and the spray gun were below the average measured breathing velocity of 5.66 ± 1.57 m/s (mean ± standard deviation), while the GloGerm^TM^ MIST velocity was above, however, the average velocities of GloGerm^TM^ MIST and the spray gun fall into the reported 2.2 m/s to 9.9 m/s range for breathing velocity [26]. In the context of cough simulation, the reported physiological cough velocity range is 6 m/s to 28 m/s and only GloGerm^TM^ MIST can produce a simulated cough within this range [27,28,29].

### 3.2. Analysis of Simulated Cough Dispersion Using 3D Reconstruction

**Figure 3:**
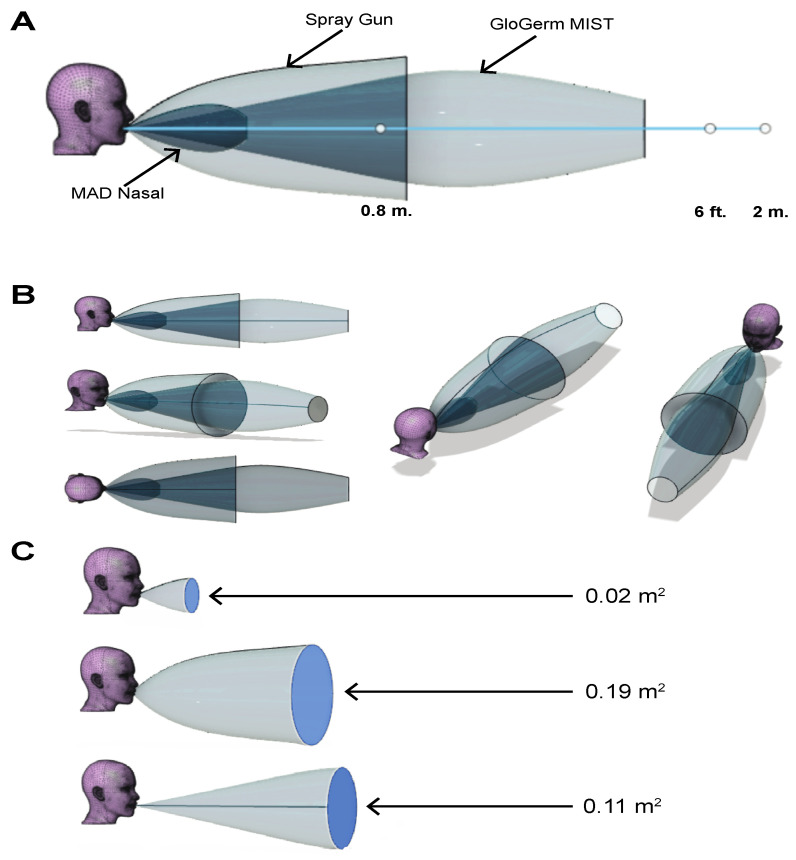
3D reconstruction of simulated coughs. (**A**) A side view of all three simulated cough 3D reconstructions is shown with the relevant distances denoted by a white dot on the light blue line. (**B**) Multiple profiles of the 3D reconstructions are shown to capture visualizations from all three dimensions. (**C**) Cross sections of each reconstructed cough on Fusion 360 is shown along with the respective maximum detectable 2D projected area.

In order to analyze the distance spread of each cough simulator, the 0.8 m and > 2 m were used as markers for how far a physiologic breath or cough travels respectively [30]. Both the spray gun (0.89 m) and GloGerm^TM^ MIST (1.62 m) meet or surpass the 0.8 m threshold for how far the droplets spread away from the cough simulator, however the MAD Nasal (0.38 m) does not reach 0.8 m. None of the simulators reach 2 m or even the CDC recommended social distancing length of 1.82 m (6 feet) with the closest simulator being GloGerm^TM^ MIST (Figure 3A). Although these thresholds have been created by the CDC and data from the literature, there is variability in the distance a simulated cough or physiological cough can travel, and the actual spread of a cough can be farther based on the duration of the cough. For example, a study that used computational fluid dynamics to analyze SARS-CoV-2 spread shows that over time, SARS-CoV-2 can spread up to 6.5 m [5]. Therefore, the spread of respiratory droplets can also occur beyond 1.82 m and 2 m depending on the scenario. During analysis of the maximum detectable 2D projected area, a reported range of 0.01 m^2^ to 0.14 m^2^ range was used for comparison [30]. Analyzing the maximum detectable 2D projected areas—or in other words the maximum area that can be covered by a cough—GloGerm^TM^ MIST (0.11 m^2^) and MAD Nasal (0.02 m^2^) are the only simulators that fall in the reported range, while the spray gun produces a maximum detectable 2D projected area (0.19 m^2^) outside the upper limit of the reported range (Figure 3C).

### 3.3. Simulated Cough Force Analysis

**Figure 4:**
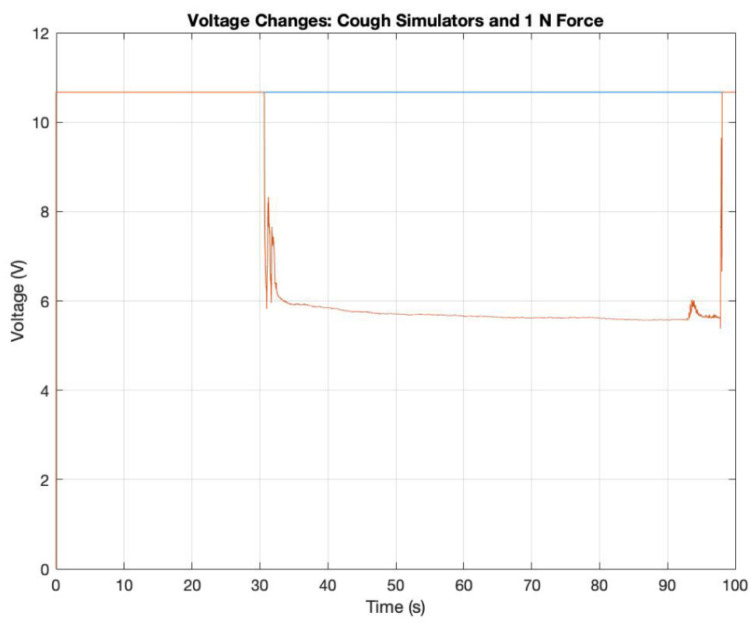
Force analysis of simulated coughs. A time-dependent graph of voltage that was captured using MATLAB during the force measuring experiment as all three simulated coughs, a human breath, and a human cough, were applied to the force-sensing resistor in the first 25 s with no voltage change and a contrast 1 N standard weight applied at 30 s.

Our force analysis revealed that the force produced by each simulator, a human breath, and a human cough was <0.5 N, which is the threshold force needed to detect a change in voltage for our force-sensing resistor. No changes in voltage were recorded from the force-sensing resistor during these activities (Figure 4). A standard weight of 1 N is shown to contrast with the steady voltage of 10.688 V in the circuit.

## 4. Discussion

In this study, we characterized various properties of commonly utilized cough simulators that are used to study how respiratory droplets can spread in clinical encounters. These cough simulators have been used in a number of recent reports, in particular to model SARS-CoV-2 viral droplet dispersion [10,12,13,14,15,17,20]. Since these simulations are used to inform clinical guidelines for PPE, it is important to understand the physiological accuracy of these simulators. Here we show that the droplet sizes produced by MAD Nasal, a spray gun, GloGerm^TM^ MIST, and a Phillips nebulizer are all >100 µm and fall into the large respiratory droplet category and not into the small respiratory droplet category of 5–10 µm. The average simulated cough velocity of the spray gun and GloGerm^TM^ MIST fall in the range for human breathing velocity, and GloGerm^TM^ MIST is the only simulator to fall in the velocity range for human coughing. Likely explanations of this deviance in droplet size distribution include the differences in viscosity of the surrogate droplets compared to physiological droplets and the inherent limitations of the devices used to create the droplets, such as the nozzle sizes which will influence the liquid film fragmentation, aggregation of droplets on the carbon tape, and the distance at which the simulated cough was delivered to the carbon tape. Deviances in the droplet size also help to explain the difference between simulated cough velocity and physiological cough velocity, but a more chemo-physical investigation is needed to provide a solid explanation.

3D analysis shows that MAD Nasal and GloGerm^TM^ MIST can cover an area with droplets within the reported range for human coughing, but MAD Nasal cannot produce droplets that travel far enough to be comparable to human breathing or coughing. In fact, while GloGerm^TM^ MIST and a spray gun can eject droplets as far as human breathing, none of the simulators can eject droplets as far as a human cough with GloGerm^TM^ MIST producing the farthest traveling particles. Lastly, our force analysis shows that all of the simulators produce coughs with forces less than 0.5 N, however the exact force was not determined.

From all the experiments and modeling performed, it is clear that none of the abovementioned simulators accurately model all the properties of physiologic coughing or breathing found in the literature. The GloGerm^TM^ MIST canister, however, produces simulated coughs most close to physiological coughs with the largest discrepancy being that the droplets do not travel as far as physiologically ejected respiratory droplets during coughing (Table 1). Although this discrepancy exists, GloGerm^TM^ MIST produces droplets that travel the farthest and covers a larger spectrum of human cough dispersion distance than MAD Nasal and the spray gun. Furthermore, GloGerm^TM^ MIST is the only simulator that produces droplets with velocities comparable to human coughing and captures >80% of the detectable 2D projected area range for human coughing. If GloGerm^TM^ MIST is used for simulating respiratory droplet spread via human coughing in clinical settings, it is important to understand the abovementioned limitations and note the said limitations while drawing conclusions from experiments using GloGerm^TM^ MIST. 

While MAD Nasal and a spray gun are less realistic in their simulations, they can still be used for certain scenarios. For example, MAD Nasal could be ideal to track droplet spread from a neonatal or weak cough, but further evaluation would need to be done in order to confirm this. Studies that have used MAD Nasal as a cough simulator to determine how droplets will spread, need to be re-evaluated or considered as an underestimation when they are used to make the best clinical models. Moreover, the spray gun is the only cough simulator with an average velocity within the velocity range of physiological breathing and has an overlap with the reported average physiological breathing velocity within 1.5 standard deviations. In addition, the distance traveled by spray gun droplets is the closest to the reported distance traveled by physiologically expelled respiratory droplets during breathing. Overall, a spray gun is the closest in exhibiting properties similar to human breathing, but if it is used in experiments, the conclusions from those experiments need to take into account the discrepancies between a spray gun and human breathing properties.

The standards used in this study to compare simulated coughs to physiological coughs were created by doing a literature search. However, there are studies that have concluded that physiological coughs can have a different spread compared with the standards used in this study. For example, a recent publication that used computational fluid dynamics specifically to analyze the respiratory droplet and aerosol ejection for SARS-CoV-2 shows that over time a cough can spread up to 6.5 m within three seconds. None of the cough simulators examined spread close to 6.5 m, with GloGerm^TM^ MIST having the closest resemblance [5]. Therefore, while using GloGerm^TM^ MIST researchers should account for this discrepancy and can evaluate their findings based on a mild intensity cough, their analysis also shows the ejection velocity to be around 10 m/s, which is consistent with the standards used in this paper.

While we report several properties of simulated coughs and breaths and compare them to their physiological equivalents, it is important to note that some of the parameters in the experiment will vary. For example, spray guns can be adjusted to change where the droplets are concentrated, and the pressure within the GloGerm^TM^ MIST canister will drop after several sprays, meaning the canister will need to be repressured between simulations. Both of these variances can cause the droplet sizes and velocities to vary, but our mean and standard deviation capture a comprehensive range of data that should provide representative measurements. Our analysis could use further considerations, such as a more precise evaluation of the forces generated by the simulated coughs and the quantification of flow rate to provide more information regarding the physiological accuracy of such simulators. Furthermore, since the 3D reconstructions are approximations, it must also be assumed that droplets can still exist below the 3D reconstruction due to the force of gravity.

More control over physiological parameters can be attained using more expensive equipment. For example, one study used a nebulizer connected to an AMBU bag in order to create physiological tidal volume and flow rate, however, an expensive and not readily available optical particle counter was used in their study [10]. Another cough simulator that was developed in 2013 utilized a micropump nebulizer to aerosolize cell culture media and deliver a fixed flow of aerosol per unit time [11]. Although both these systems are very physiologically accurate, they are expensive, not readily accessible, and do not allow visualization of particle spread like the three simulators we evaluated.

## 5. Conclusions

We can state that based on our results, GloGerm^TM^ MIST is the most physiologically relevant cough simulator based on droplet size, 3D spread, velocity, and force of the three low-cost simulators that were studied. However, since there are dissimilarities between all three low-cost cough simulators and physiological coughing, all studies using any of the cough simulators studied here should take caution when drawing conclusions from these cough simulators. As physicians explore more effective PPE strategies, cough simulations will be used to evaluate and refine their PPE. Therefore, it is important that they can access quantitative data to determine how much these simulators physiologically resemble coughing and breathing. Moreover, the 3D reconstructions we have provided in the Supplementary Materials allow scientists to replace the use of physical cough simulations with augmented reality visualizations or quick approximations of respiratory droplet spread based on the 3D reconstruction dimensions. As each simulation can only approximate these physiologic processes, clinicians and scientists should consider these caveats when interpreting data from cough simulators.

## Figures and Tables

**Table 1:** Cough simulator comparison. Each cough simulator is given a + or − for each parameter, indicating that the simulator either meets the physiological criteria or does not. A +/− is given if the cough simulator meets some but not all of the criteria.

Cough Simulator	Droplet Size	Velocity	3D Spread	Force
MAD Nasal	+/−	−	−	+
GloGerm^TM^ MIST	+/−	+	+/−	+
Spray Gun	+/−	−	+/−	+

## Supplementary Materials

The following are available online at https://www.mdpi.com/2077-0383/9/9/3002/s1, Figure S1: Droplet Size Analysis with Caliper Measurements, Figure S2: Reconstructed Cough for MAD Nasal, Figure S3: Reconstructed Cough for GloGerm^TM^ MIST, Figure S4. Reconstructed Cough for the Spray Gun, Movie S1. Simulated Coughs Used for Velocity and 3D Analysis.
