# Supplementary material for: Assessing the Physiological Relevance of Cough Simulators for Respiratory Droplet Dispersion
Source: J Clin Med. 2020 Sep 17;9(9):3002. doi: 10.3390/jcm9093002 (PMC7564804; doi:10.3390/jcm9093002)
Supplement: Supplementary file 1 [file jcm-09-03002-s001.zip › Supplemental Material/Supplementary Figure Captions.pdf]

Supplementary Material

Movie S1. Simulated Coughs Used for Velocity and 3D Analysis
Multiple simulated coughs from each cough simulator are shown. These simulated coughs were used for the velocity analysis and 3D reconstruction.
Figure S1. Droplet Size Analysis with Caliper Measurements. The direct measurement of each droplet using the caliper function on the SEM is shown for each cough simulator.
Figure S2. Reconstructed Cough for MAD Nasal
A 3D file (.stl) is provided for the 3D reconstruction of a simulated cough produced by MAD Nasal.
Figure S3. Reconstructed Cough for GloGerm™ MIST
A 3D file (.stl) is provided for the 3D reconstruction of a simulated cough produced by GloGerm™ MIST.
Figure S4. Reconstructed Cough for the Spray Gun
A 3D file (.stl) is provided for the 3D reconstruction of a simulated cough produced by the spray gun.
