# Supplementary figures and images for: Assessing the Physiological Relevance of Cough Simulators for Respiratory Droplet Dispersion
Source: J Clin Med. 2020 Sep 17;9(9):3002. doi: 10.3390/jcm9093002 (PMC7564804; doi:10.3390/jcm9093002)

MAD Nasal

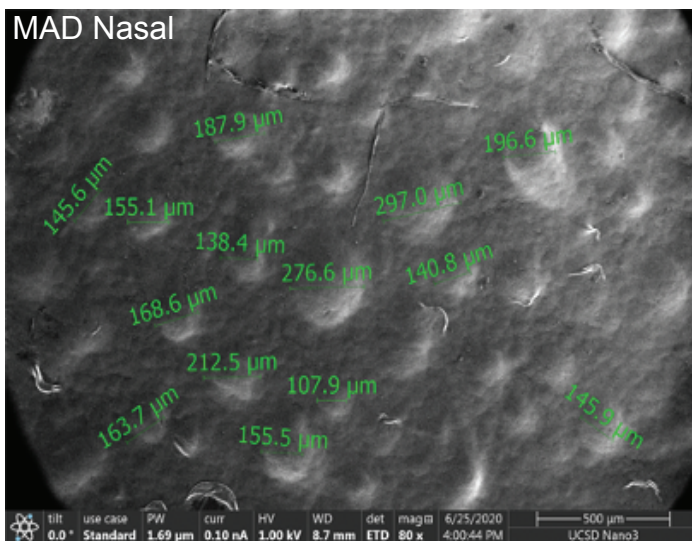

Spray Gun

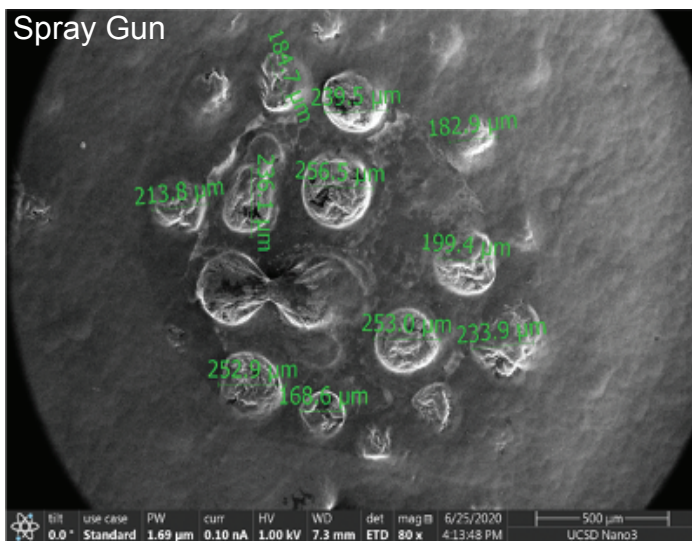

GloGerm™ MIST

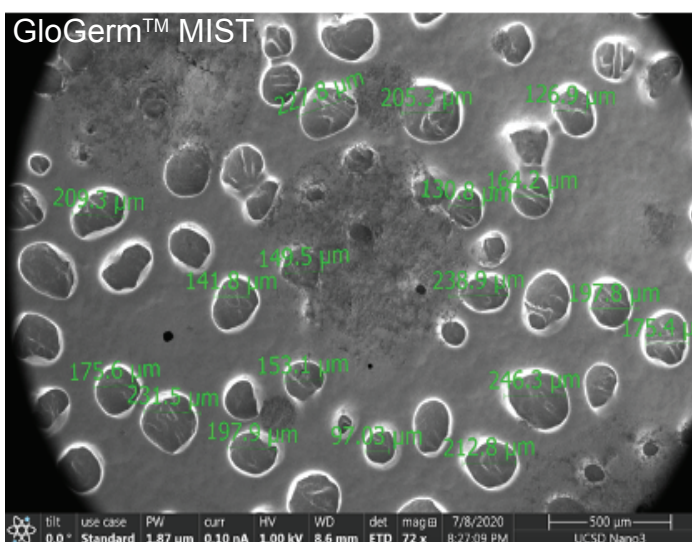

Nebulizer

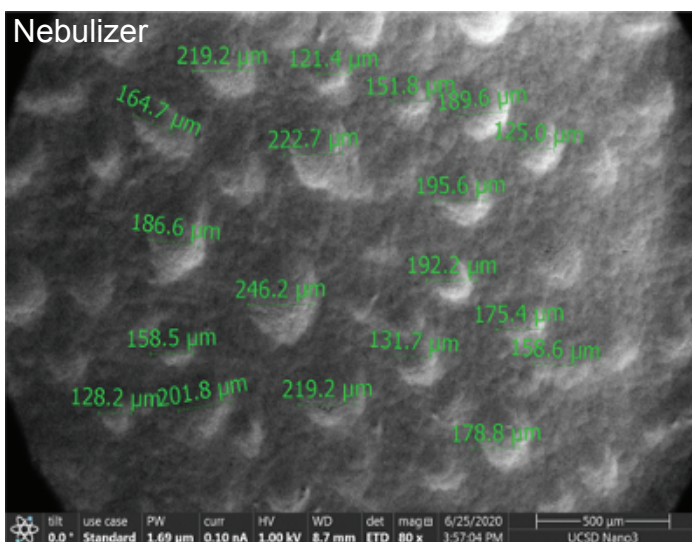

Supplement: Supplementary file 1 [file jcm-09-03002-s001.zip › Supplemental Material/Figure S1.pdf]

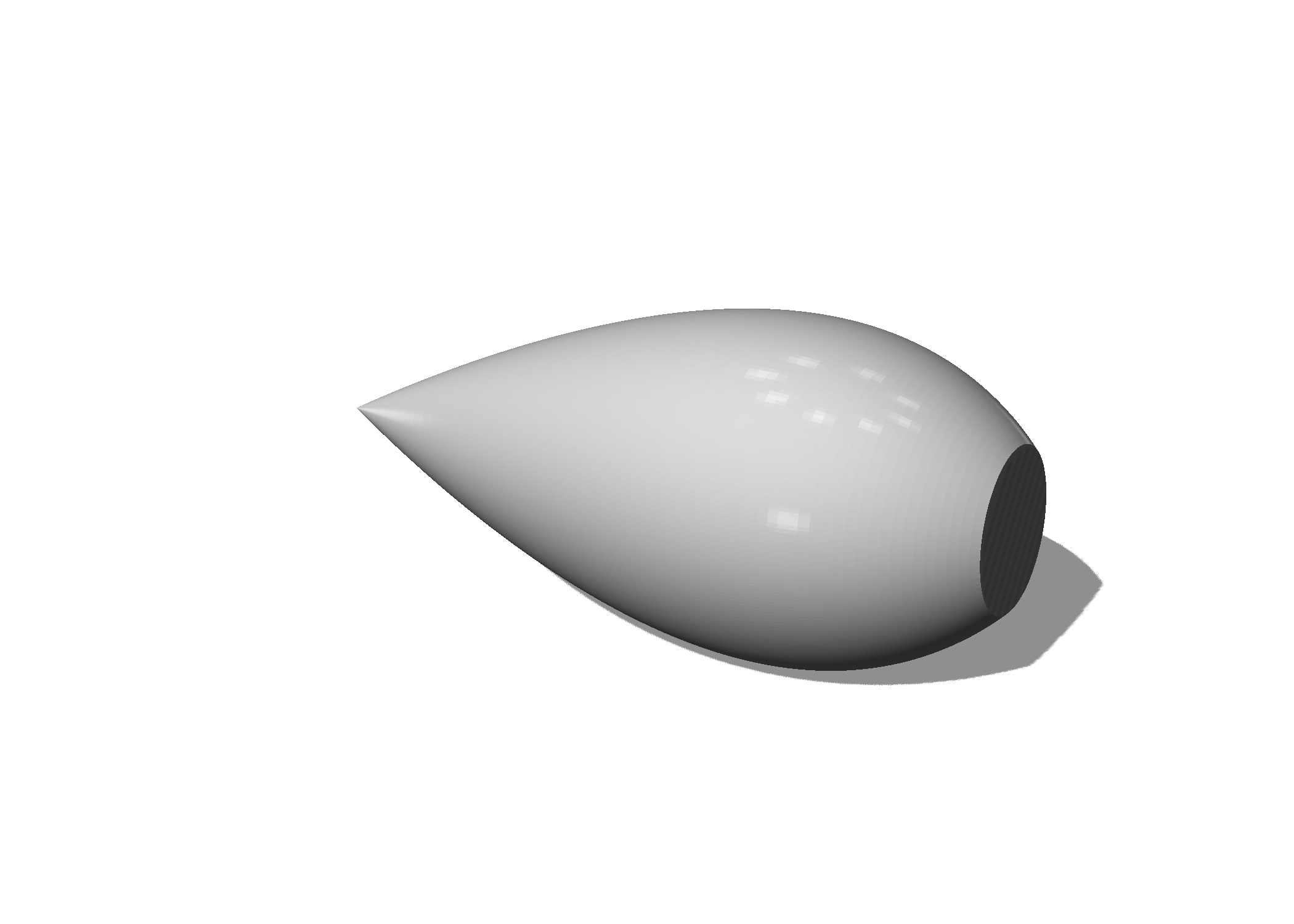

Supplement: Supplementary file 1 [file jcm-09-03002-s001.zip › Supplemental Material/Figure S2.tif]

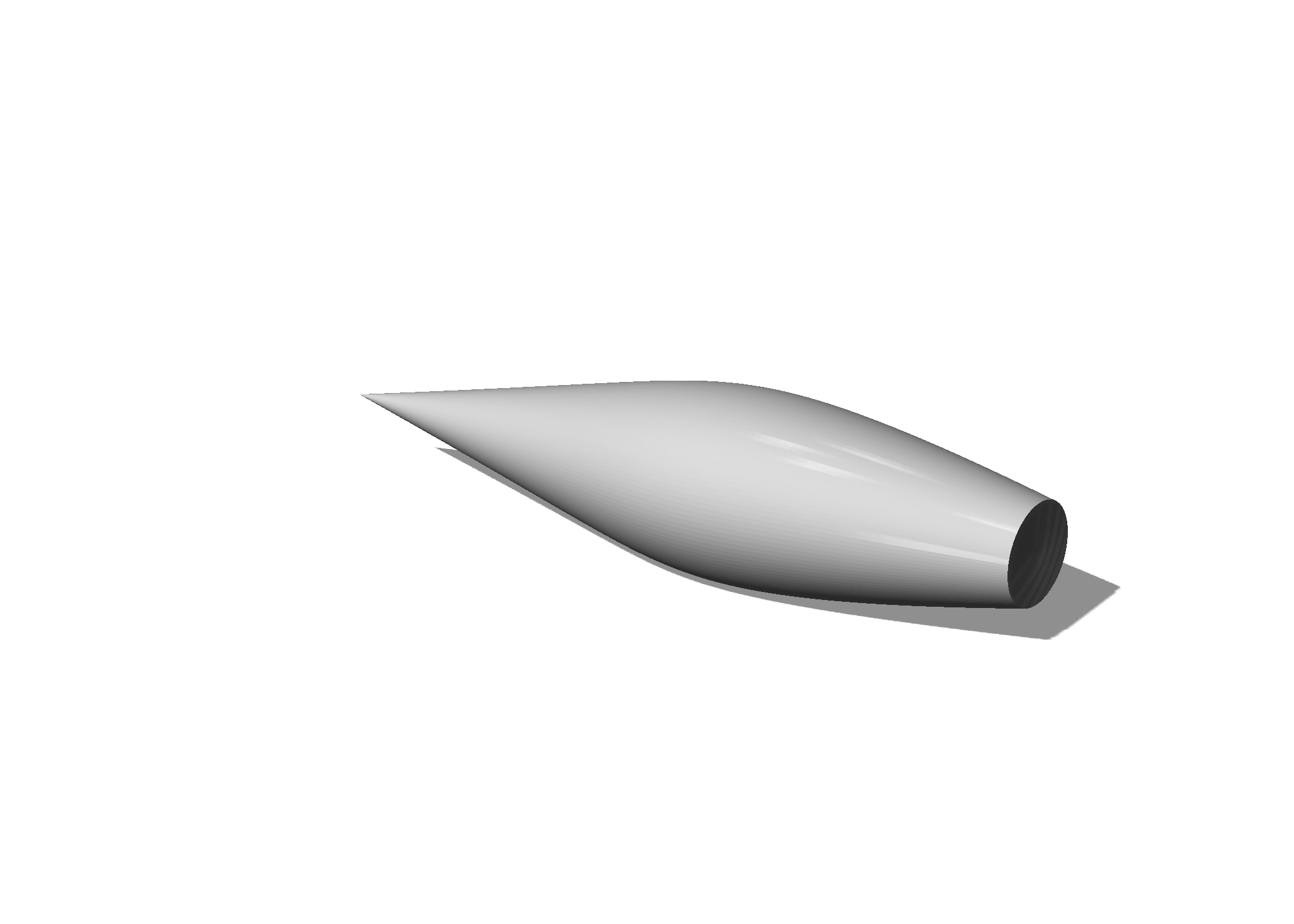

Supplement: Supplementary file 1 [file jcm-09-03002-s001.zip › Supplemental Material/Figure S3.tif]

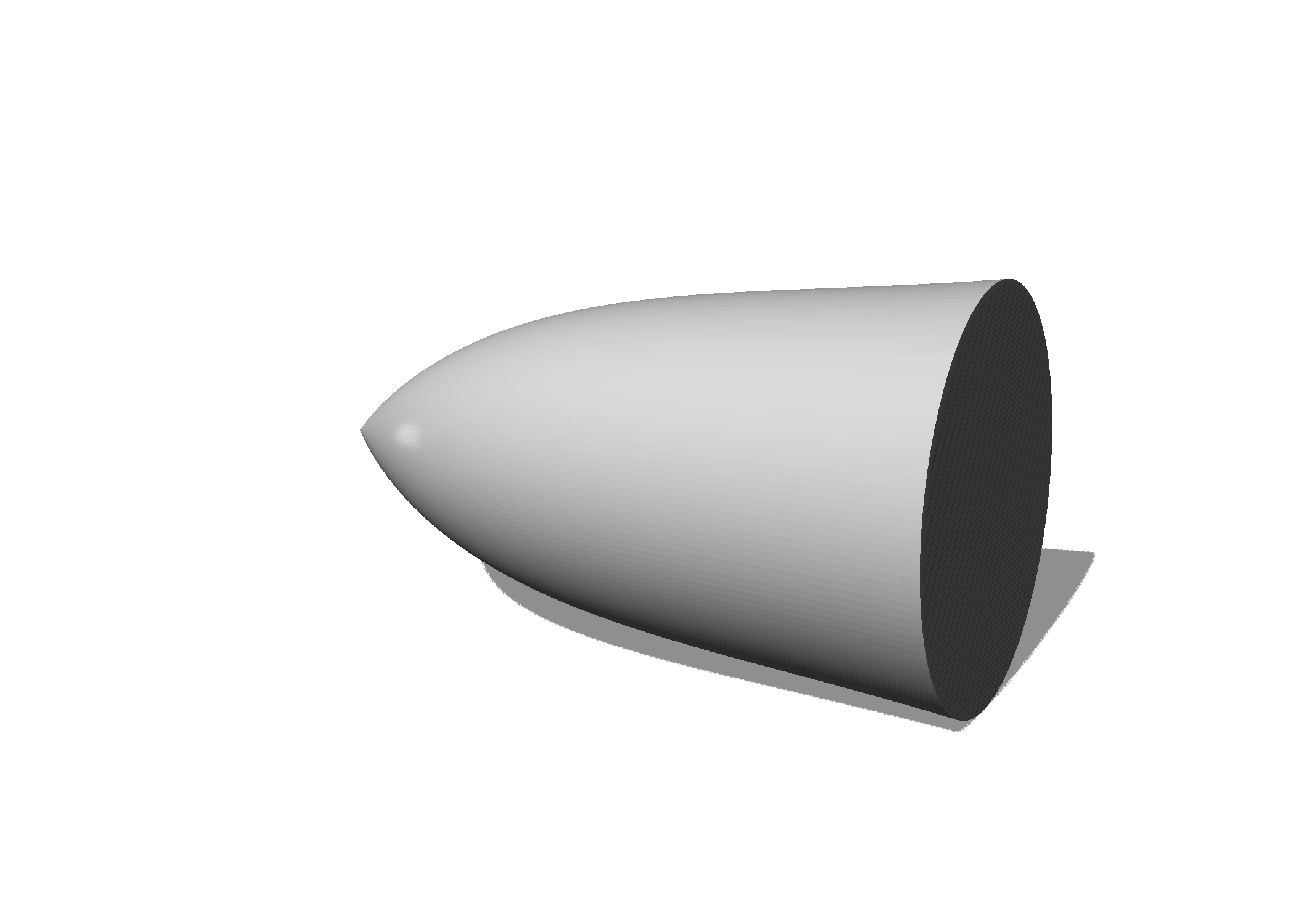

Supplement: Supplementary file 1 [file jcm-09-03002-s001.zip › Supplemental Material/Figure S4.tif]
